# Effect of Retrofit Design Modifications on the Macroturbulence of a Three-Phase Flotation Tank—Flow Characterization Using Positron Emission Particle Tracking (PEPT)

**DOI:** 10.1021/acs.iecr.2c04389

**Published:** 2023-05-03

**Authors:** Katie Cole, Diego Mesa, Michael van Heerden, Pablo R. Brito-Parada

**Affiliations:** † Department of Physics, University of Cape Town, Rondebosch 7700, South Africa; ‡ Advanced Mineral Processing Research Group, Royal School of Mines, Imperial College London, South Kensington, London SW7 2BX, United Kingdom; ¶ iThemba LABS, Old Faure Road, Eerste River, Cape Town 7100, South Africa

## Abstract

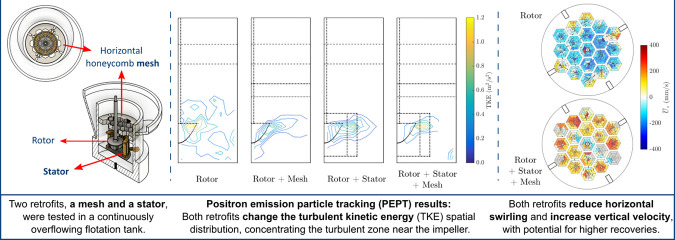

Turbulence in stirred tank flotation tanks impacts the bulk transport of particles and has an important role in particle–bubble collisions. These collisions are necessary for attachment, which is the main physicochemical mechanism enabling the separation of valuable minerals from ore in froth flotation. Modifications to the turbulence profile in a flotation tank, therefore, can result in improvements in flotation performance. This work characterized the effect of two retrofit design modifications, a stator system and a horizontal baffle, on the particle dynamics of a laboratory-scale flotation tank. The flow profiles, residence time distributions, and macroturbulent kinetic energy distributions were derived from positron emission particle tracking (PEPT) measurements of tracer particles representing valuable (hydrophobic) mineral particles in flotation. The results show that the use of both retrofit design modifications together improves recovery by increasing the rise velocity of valuable particles and decreasing turbulent kinetic energy in the quiescent zone and at the pulp–froth interface.

## Introduction

Froth flotation is the most important mineral concentration method in the mining industry. Mechanical flotation tanks are the predominant equipment used for this mineral concentration method and are characterized by an agitation mechanism that generates a turbulent flow regime. Turbulence has a key role in the particle–bubble interactions of flotation on different length scales. It suspends particles in the main flow profile of the tank on the macroscale and causes bubble breakup and particle–bubble collisions on the microscale.^1^ Models of these microprocesses suggest that attachment occurs in areas of high energy dissipation, such as in the impeller discharge stream.^1^

### Flotation Tank Design and Retrofit Design Modifications

The design of a flotation tank, including its geometry, agitation mechanism and air distribution system, plays a significant role in the fluid dynamics in the pulp and froth zones.^2,3^ Physical modifications of the flotation tank design, therefore, affect the attachment efficiency and thus impact the metallurgical performance of flotation. As an example, an industrial study by Tabosa et al.^4^ found that there was a correlation between tank size and froth recovery, with a short aspect ratio tank having 40 to 50% lower froth recovery due to the high turbulence levels near the pulp–froth interface, and a long aspect ratio tank having too deep a quiescent zone, which led to a low flotation rate. The addition of larger impeller mechanisms and retrofit design modifications to flotation tanks, such as horizontal rings or turbulent diffusers, can optimize the distribution of turbulence and increase the rate of flotation.^4^

A retrofit design modification corresponds to an insert that can be added to the tank after installation in order to modify the flotation phenomena, hereafter abbreviated as “retrofits”. The main advantage of these retrofits is that they bring improvements to flotation performance at a reduced capital expenditure, without having to install new flotation tanks at prohibitive expense. Launders and crowders are common examples of retrofits for the froth zone, as reviewed by Mesa and Brito-Parada^5^ and Jera and Bhondayi.^6^

Although retrofits have been widely incorporated into industrial operations and there are several studies of their effect on flotation performance,^7−10^ the influence of retrofit designs on turbulence has not yet been explored. This gap in the literature may relate to the technical challenges associated with the measurement of the turbulent fluid dynamics of opaque multiphase systems.

### Positron Emission Particle Tracking Measurements of Turbulence

In this work, positron emission particle tracking (PEPT) was used to characterize the effect of two retrofits on the particle dynamics of a flotation tank. PEPT offers the advantage of being able to track particle flow in opaque, three phase systems, whereas other tracking techniques such as particle image velocimetry (PIV) tend to be restricted to single or two phase systems.^11,12^ Since its first application to froth flotation by Waters et al.,^13^ PEPT has been established as an important technique for studying the fluid dynamics of flotation systems. Recent applications of PEPT to flotation include the study of coarse particle flotation,^2,14,15^ normal operating size limits^16^ and individual bubble–particle interactions.^17^

Until recently, the turbulent properties of fluid behavior were not commonly calculated from PEPT data. Gabriele et al.^18^ and Liu and Barigou^19^ suggest several reasons for this absence as arising from experimental limitations, such as (i) the large size of the tracer particles relative to the size of turbulent structures, (ii) the length of the experiment required to obtain statistical significance on calculated kinematic values (see also Windows-Yule^20^), (iii) the accuracy required in locating the tracer particle with respect to the positron emission tomography (PET) camera used and the data methods, and (iv) the relatively short half-life of the radionuclides used for tracer fabrication. Wiggins et al.^21^ were able to overcome some of these limitations with numerous advances in multiple particle tracking techniques to characterize the turbulent stresses in water flowing through a pipe. The “M-PEPT” tests required new tracer location procedures^22,23^ calibrated with simulations of the measurements,^24^ the consistent radiolabeling of over 300 tracer particles of size 90 μm with the radionuclide ^18^F and the use a small animal PET scanner with high geometrical detection efficiency.^21^ These tools were later extended to explore the structures of vortices with a twisted tape insert, for application in the nuclear energy sector.^25^ Savari et al.^26^ used a multiscale wavelet analysis to decompose the Lagrangian trajectories derived from PEPT measurements of a particle in a single phase stirred tank reactor to extract different parts of the particle behavior in the frequency domain. Measurements of the fluid turbulent kinetic energy (TKE) in different profiles of the reactor were validated with PIV measurements to provide further information on turbulent diffusion in the fluid. These methods of utilizing the Lagrangian trajectories were extended to find flow structures in single and solid–liquid two phase stirred tank reactors from Lyapunov exponent analysis.^27^

For our own measurements with PEPT of particle motion in three phase froth flotation, the calculation of TKE experienced by a PEPT tracer particle has become feasible for turbulent structures at macroscale length scales due to several improvements. The application of the Siemens “HR++” PET scanner at PEPT Cape Town^28^ with high geometric detection efficiency in combination with careful data treatment^2,29^ and new tracer techniques^30,31^ has enabled comparison of the velocity fields of different solid and fluid tracer particles and the resolving of flow structures of several millimeters in size.^14^ However, it remains to be determined whether PEPT can track the highest frequency components of the fluctuating path of the tracer particle and resolve the structure of turbulent flow on the microscale due to the inherent sources of noise in a PEPT measurement, such as the finite range of the positron before annihilation, which leads to an uncertainty in the tracer position in each location measurement.

The evaluation of the uncertainty in a PEPT measurement is an emerging field of research. Sources of uncertainty include the slight accolinearity of the photon pair, the range of the positron in local media and the geometric detection efficiency of the field of view of the PET scanner;^32^ however, it is an area of development to establish how these sources contribute to the overall uncertainty in a location measurement performed with the HR++ scanner at PEPT Cape Town.^33^ Based on measurements of stationary and moving sources, Buffler et al.^28^ determined the uncertainty in a PEPT measurement with the HR++ to be on the order of 1 mm, which agrees with uncertainty values from Cole et al.,^29^ derived from tracking a particle moving on an impeller tip. Ultimately, the future application of Monte Carlo simulations in the software GATE (GEANT 4 Application for Tomographic Emission) will be used to explore the uncertainty in a PEPT measurement, based on published examples for different scanners and granular flow experiments.^34−36^

In this work, profiles of the particle flow dynamics, residence time and turbulent kinetic energy were derived from PEPT measurements of hydrophobic particles in a flotation tank fitted with two different retrofits: a rotor-stator impeller mechanism (after the design of Mesa et al.^2^) and a honeycomb mesh with a blocked perimeter (after the design of Mackay et al.^37^).

### Stator Retrofit

The first retrofit examined in this study is a stator. A rotor-stator mechanism is composed of a driven mixing element, the radial impeller, in close proximity to a fixed mixing element, the stator.^38^ The rotor-stator system increases the effective impeller size, which creates high shear rates and turbulence in the flow inside the stator.

Although stators have usually been regarded as a feature of the agitation mechanism, they can be studied as a retrofit to the pulp-zone, as they can be easily replaced by novel designs over the lifetime of a flotation tank. An example of this kind of stator is the one used in the Outotec “OK” rotor-stator system. In the OK system, the stator is mounted on a pedestal outside the impeller^39,40^ and can be exchanged with new stator designs. Several authors^41−43^ suggest that this rotor-stator system was designed to increase mixing for a given air rate, increase the suspension of coarse particles in comparison to a conventional impeller design, and achieve a balance between hydrodynamic and static pressures in the pulp. The system results in a uniform dispersion of air over the surface of the blades and provides separate zones for the distribution of air and the pumping of slurry. In terms of flotation performance, Schubert^1^ found that the rotor-stator system influenced the entrainment of fine particles. In terms of bubble size, Mesa and Brito-Parada^44^ found that the stator reduced the Sauter mean bubble size and suggested that the stator increases turbulence and bubble breakup locally and prevents large bubbles from leaving the stator. In terms of flow dynamics, Harris^45^ suggested the baffles or blades of the stator reduce the horizontal swirl of the pulp. Recently Mesa et al.^2^ compared the fluid dynamics of different impeller-stator combinations with PEPT and found that the stator modifies the flow patterns in the tank by reducing the flow velocity outside the stator, reducing the rotational motion of the pulp and froth and generating a deeper and more quiescent froth zone. Moreover, Mesa et al.^3^ showed that the inclusion of a stator enhanced froth stability and improved flotation performance.

### Horizontal Baffle Retrofit

The second retrofit examined in this study is a horizontal baffle. Horizontal baffles, or meshes, are examples of retrofits to the vicinity of the pulp–froth interface that reduce turbulence.^46^ These retrofits promote a more quiescent pulp–froth interface and a more stable froth, both of which are associated with improved metallurgical performance. Norori-McCormac^47^ installed meshes with a honeycomb structure in a 4 L laboratory scale flotation tank operating with a single species silica system and found, using flotation tests and PEPT measurements, that meshes increased the mass flow rate of solids in the concentrate but not the concentrate grade. The PEPT measurements showed a horizontal “swirling” motion in the pathlines above the interface in the cases without a mesh, which could be the cause of low froth stability. This swirling motion was reduced by the presence of the mesh, which suggests that the mesh decoupled the pulp from the froth. Morrison^48^ installed square meshes on a larger tank for scale-up (87 L) that led to an improvement in recovery and grade but not air recovery. Mackay^49^ used a synthetic magnetite and silica system and found that a honeycomb mesh design with a blocked section around the perimeter facing the wall of the tank led to improved recovery and less entrainment. Mackay^49^ also completed PEPT measurements of hydrophilic and hydrophobic particles, which showed that the mesh caused a reduction in the horizontal swirl of the froth. A secondary circulation loop of pulp formed above the mesh in some configurations, which may have promoted reattachment as the system recovery was higher.

### Scope of the Work

The main contributions of this work to the field of retrofit design for flotation tanks are presented as follows: (i) the comparison of the effects that retrofit design configurations can have on particle dynamics in flotation, (ii) the calculation of TKE for an opaque, multiphase and polydispersed turbulent system, and (iii) an extensive empirical database of three phase fluid dynamic behavior that is useful for the validation of computational models and simulations for future improvements in industrial scale flotation efficiency.

## Materials and Methods

### Flotation Tests

The flotation tank used for PEPT experiments has been fully presented by Mesa et al.,^2^ which is based on the design presented by Norori-McCormac et al.^50^ The proportions of the tank were based on the standard configuration (after Rushton et al.^51^ and Eckert et al.^52^), with a diameter and height equal to 180 mm. The tank contained four vertical baffles up to a height of 120 mm. It was agitated using a rotor impeller based on the Outotec OK design,^40^ of diameter 60 mm and driving frequency 1200 rpm, as described by Mesa et al.^2^

An external launder enabled continuous operation of the tank as the overflowed concentrate was recycled to the base of the pulp with a peristaltic pump. An air rate corresponding to a superficial gas velocity of 0.98 cm/s produced fine bubbles through a sintered steel mesh plate. The solids loading was composed of a single species of spherical glass beads particles of diameter 75 to 150 μm and a concentration of 20.0%_*w*,*w*_. The particle size distribution of a representative sample of the glass beads is given in the Supporting Information, Figure S2. The silica collector was myristyl trimethylammonium bromide (TTAB) at 4.0 g TTAB/t_SiO_2__, and the frother was methyl isobutyl carbinol (MIBC) at water volume concentration 4 ppm. The pH was not controlled, as flotation was not optimized in this experiment. For further experimental details, see reports of similar experiments in Mesa et al.,^2^ Cole et al.,^29^ and Mesa et al.^31^

**Figure 1:**
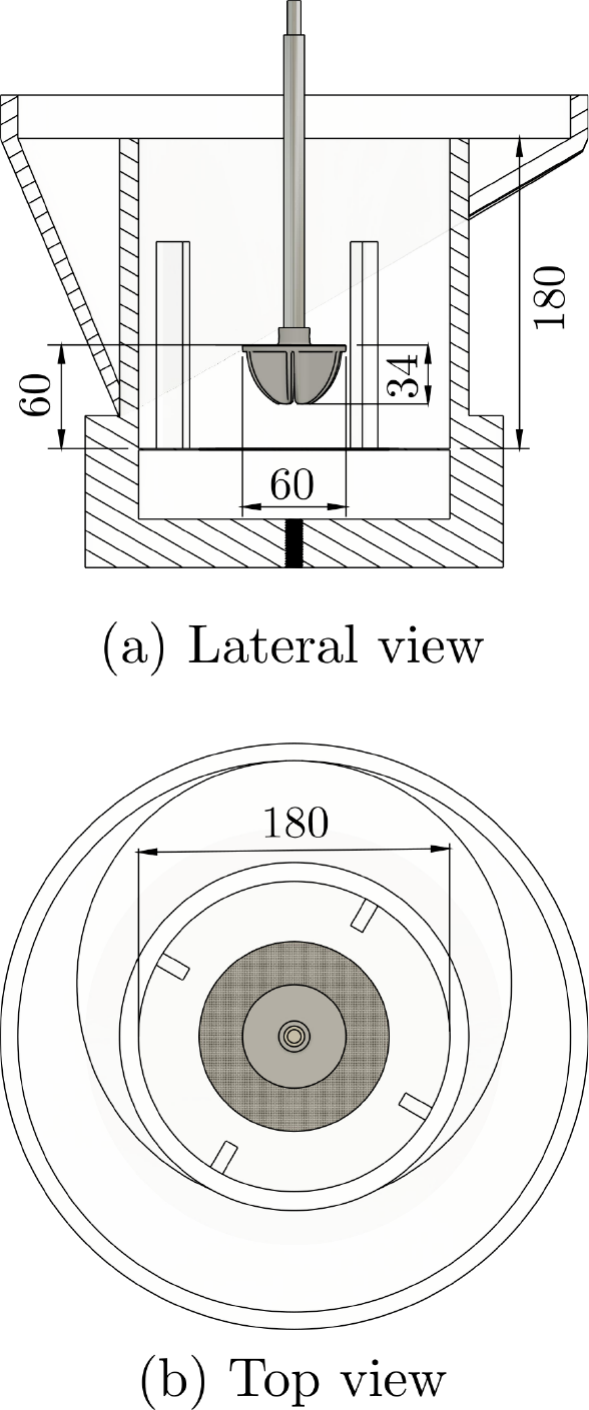
Aerated stirred tank for flotation, showing the dimensions from (a) lateral and (b) top views. The tank is fitted with a rotor of 60 mm diameter, and its proportions follow that of a standard mixed tank.

**Figure 2:**
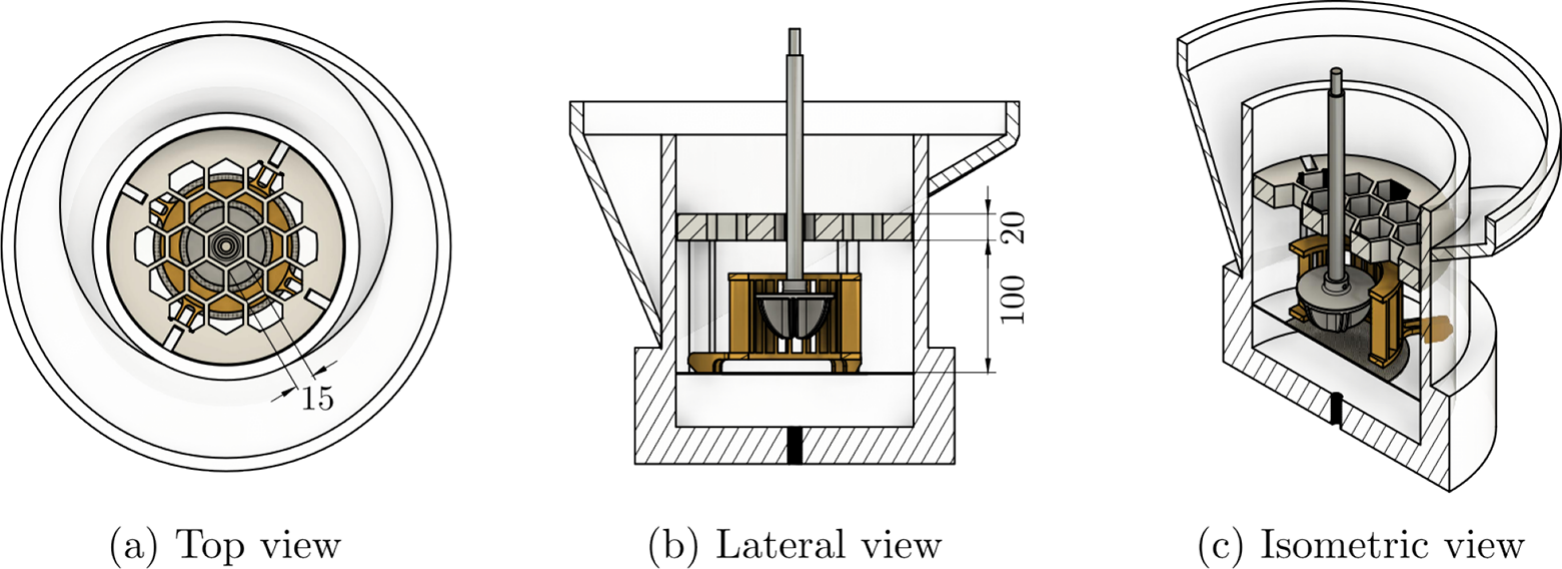
Configuration of the bench-scale flotation tank as fitted with a rotor-stator impeller mechanism and a horizontal mesh, showing the dimensions from (a) top, (b) lateral and (c) isometric views.

Two retrofits were tested with the rotor impeller to study their effect on turbulence. The retrofits studied were a stator of 20 blades, as designed by Mesa and Brito-Parada,^44^ and a honeycomb mesh with a blocked perimeter, as shown by Mackay,^49^ to increase recovery and decrease entrainment in a laboratory scale flotation tank. The flotation experiment was repeated four times, corresponding to the combinations of rotor, rotor + stator, rotor + mesh, and rotor + stator + mesh. The dimensions of the flotation tank and the configuration of the retrofit devices are shown in Figures 1 and 2. For radiation safety, any interaction with the slurry during PEPT experiments was avoided, as there is a small probability that the tracer could break up at any time and leach its radiolabeled activity into the liquid, where it tends to selectively adsorb onto the silica surfaces. Consequently, the effect of the retrofit designs on the metallurgical performance was not part of the scope and will be considered in a future work. Previous works^44,47−49^ have shown that these retrofits can improve the froth stability and metallurgical performance of a flotation cell.

### PEPT Methodology

The flotation tank was positioned in the center of the field of view (FOV) of the PET camera (ECAT HR++, model CTI/Siemens 966) at PEPT Cape Town, which is located at iThemba LABS in South Africa.^28,53^ A single hydrophobic tracer particle was produced for each vessel configuration with a different retrofit design modification. The hydrophobic tracer particles used for each experiment had initial activities ranging from 750 to 1500 μCi (27.8 to 59.2 MBq) of the radionuclide ^68^Ga radiolabeled on the surface of an ion-exchange resin. The tracers had a coating of hydrophobic silica using epoxy resin adhesive, which resulted in an ellipsoid shape with minor and major axes of 500 and 600 μm, respectively.^30^

The location data from the pulp (*X*, *Y*, *Z*; *t*) were derived using the Birmingham *track* algorithm.^54^ The bin size, *N*, was calculated from the average number of lines recorded per 1 ms to correspond to a location frequency of approximately 1 kHz and a final fraction, *f*, of 0.30.^29^ The value of *N* was reduced linearly with tracer activity (exponentially with time) to account for the decreasing number of lines recorded due to the decay of ^68^Ga over each experiment,^55,56^ down to a minimum activity of 150 μCi, below which the tracer could not be reliably detected near the impeller.^29^ The location data with time were smoothed using a kernel formed of two cubic splines with a half width of Δ*t* = 4 ms to remove high frequency noise in the tracer paths.^29,57^

**Figure 3:**
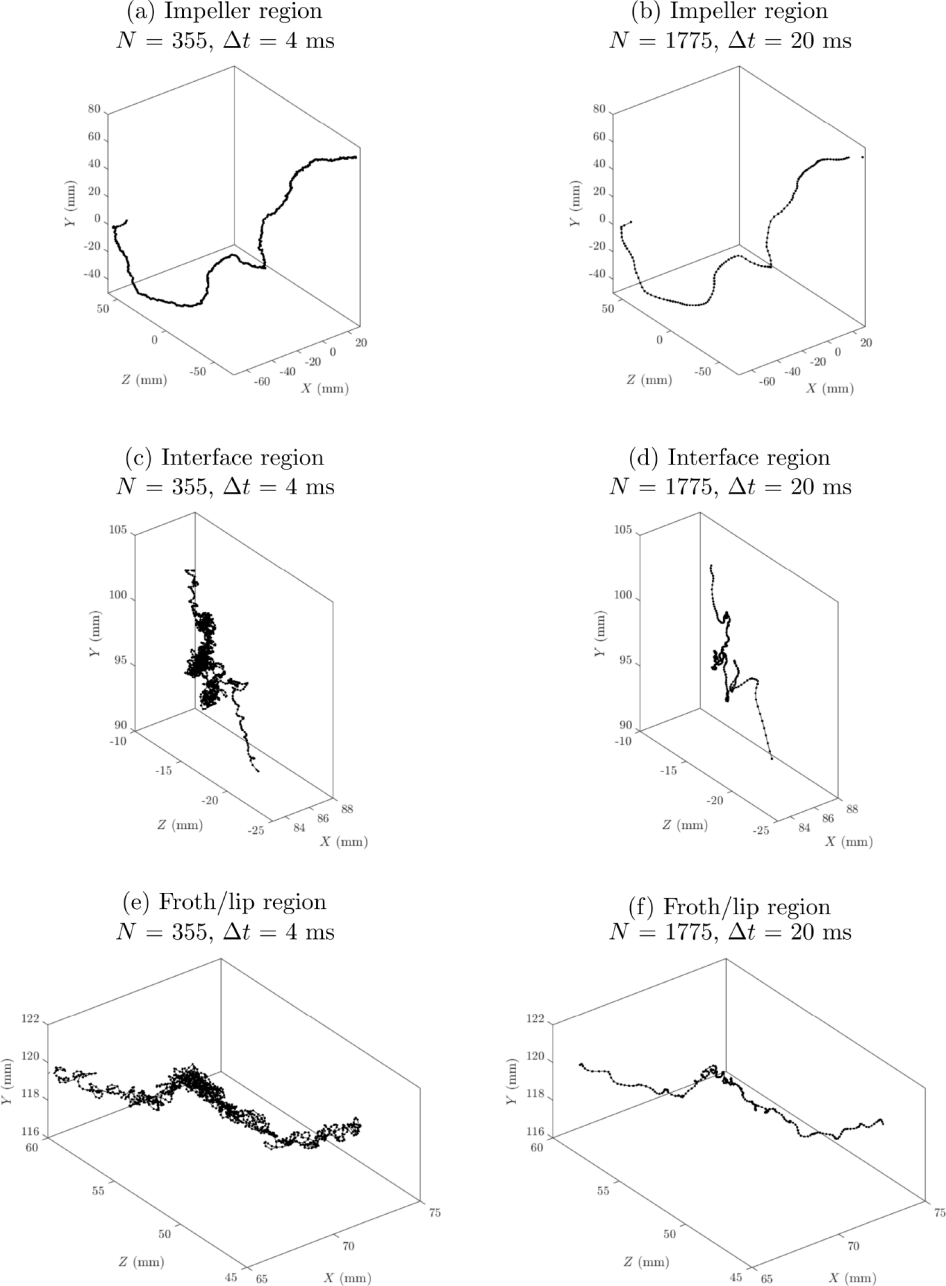
Example trajectories of duration 1.00 s of a hydrophobic particle moving in different positions around the tank as derived with two location schemes: (a, b) in the pulp near the impeller, (c, d) near the interface, and (e, f) in the froth, at the lip level. The tracks on the left (a, c, e) were derived with *N* and Δ*t* and on the right (b, d, f) with 5*N* and 5Δ*t*. The coordinate system was configured with *Y* = 0 mm at the impeller plane and (*Z*, *X*) = 0 at the center of the FOV.

A second location scheme used higher values of 5*N* and 5Δ*t* as the chosen parameters for the location procedure above the mesh level to further reduce noise in the location data.^2^ The 5*N* scheme is used through the mesh and above in the froth, where the average speed of the tracer particle is slower, with an order of magnitude of 1 cm/s. A larger 5*N* means a larger number of lines of response can be used in each location calculation to reduce high frequency noise in the tracer path. The corresponding decrease in location rate does not impact the level of detail in the track in the upper region of the vessel as the tracer is moving more slowly, and path details to a millimeter scale can be resolved. Examples of the final trajectories using the two location schemes are shown in Figure 3.

For the data in the impeller, Figure 3(a), the *N* location scheme followed the high speed flow (order of magnitude 1 m/s) with a high location frequency of approximately 1 kHz. The 5*N* scheme as shown in Figure 3(b) resulted in a path with a lower location rate, approximately 0.2 kHz, which in turn resulted in the removal of smaller scale features in the flow due to the smoothing. In the example path near the interface, Figure 3(c, d), the higher location rate scheme in Figure 3(c) contains considerable high frequency noise as the location procedure is locating the particle in steps considerably smaller than the size of the particle. The higher *N* scheme in Figure 3(d) has removed most of this noise while still retaining the features of the path that starts at the lower end of the figure. This path likely represents the interaction between the tracer particle and the bubbles at the dynamic interface. In Figure 3(e, f) the trajectory is a path in the top of the froth, near the lip level, and represents a particle in an overflowing streamline. The data located at 1 kHz (Figure 3(e)) contains high frequency noise as in the example near the interface, which is removed by using higher values for *N* and Δ*t*. Both schemes, *N* and 5*N*, were combined to produce a final array for plotting with time-averaged voxel values below the mesh derived using the *N* tracks, and through the mesh and above derived using the 5*N* tracks.

A method of weighted averages over 11 adjacent locations was used to calculate the velocity of the tracer particle in different dimensions using the relationship from Stewart et al.^58^ Cylindrical polar coordinates (*r*, θ, *z*; *t*) were used to calculate the radial velocity , azimuthal velocity , and vertical velocity  of the location with time.

Around 2 h of velocity data per tracer were used to derive time averaged 3D Eulerian measurements of particle velocity in two voxel configurations. The first voxel configuration was (Δ*r*, Δ*θ*, Δ*z*) = (10 mm, 5°, 10 mm).^14^ Four azimuthal slices taken at the midpoints between the baffles were summed at spacings of 90°, using the symmetrical proportions of the tank to maximize the number of data points within each voxel. The first voxel configuration relative to the geometries of the vessel and retrofit design modifications is shown in the Supporting Information, Figure S1, including both absolute values for the slice limits and as proportions to the vessel radius *R* and height *H*. Gaussian kernel density estimation was used to find the probability density function (PDF) of the distributions of velocity components for each voxel, and the peak of each PDF was used as the modal average of the velocity.^29^

**Figure 4:**
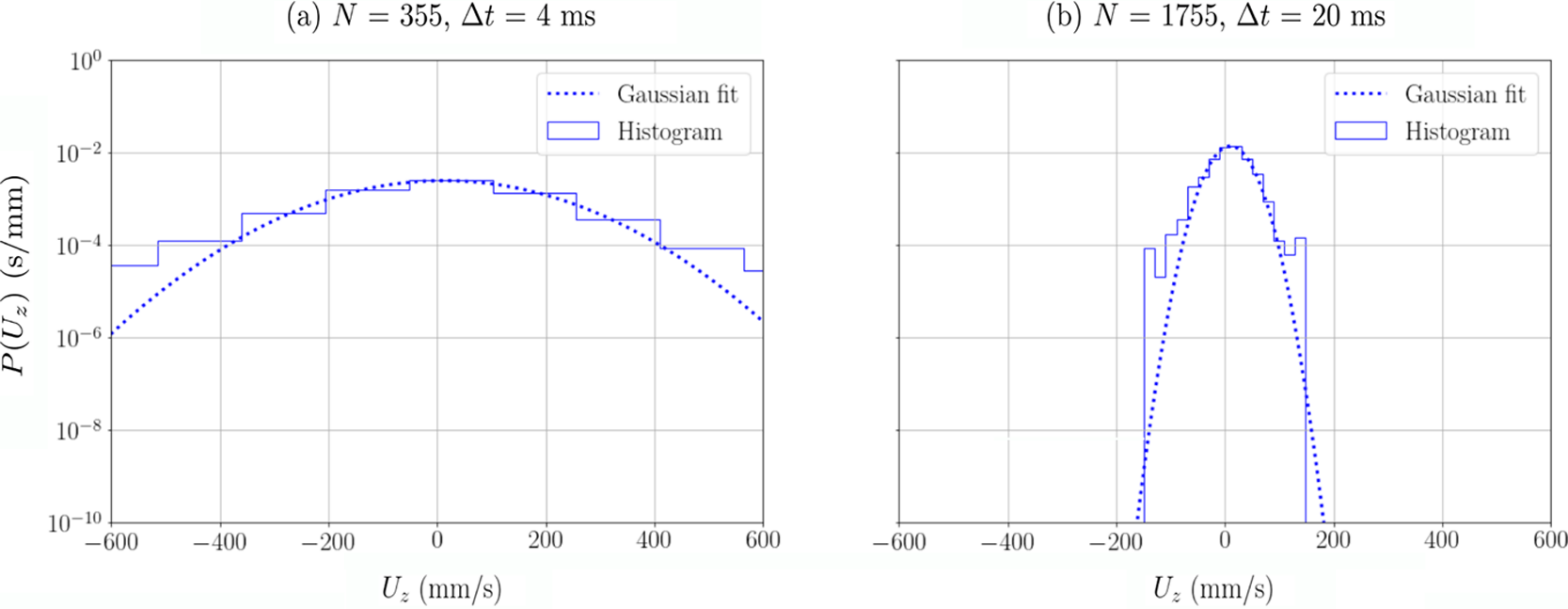
Histograms with fitted Gaussian functions of the vertical velocity of the hydrophobic particle *U*_*z*_ in a voxel in the froth processed with two different location schemes: (a) *N* = 355 and Δ*t* = 4 ms, and (b) 5*N* = 1775 and 5Δ*t* = 20 ms.

Examples of the distribution of the vertical velocity measured for the hydrophobic particle in a position in the froth are shown in Figure 4 for the two location schemes. The impact of the additional smoothing of the tracer path due to increasing *N* and Δ*t* is also illustrated in this figure, where the smoothing reduces the high frequency noise and narrows the width of the velocity histogram.

The average velocity components *U*_*r*_ and *U*_*z*_ were used to calculate azimuthal flow profiles of the tank at an angle corresponding to the midpoint of the baffles using the *streamslice* function in Matlab. Voxels were removed from the results when the distribution contained fewer than 25 data points.^29^

The residence time fraction per unit volume *T* was calculated as the total time the tracer spent in a voxel relative to the total time the tracer spent in the tank and the volume of each voxel. A linear interpolation scheme was used to find the times the tracer crossed the boundaries of each voxel.

Based on a Reynold’s decomposition, the velocity distribution in each voxel was split into an average *U*_*i*_ and a fluctuating component *u*_*i*_ in each dimension *i*,
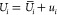
1where the fluctuations were calculated as the root mean squared difference from the peak velocity value. The turbulent kinetic energy (per unit mass)^59,60^ was calculated for each voxel as
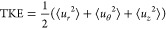
2All of the resulting data per voxel, including the velocity, fluctuating velocity component and TKE for each component, as well as the tracer trajectory data, has been compiled and compressed into a Data.zip file, available in an open-source data repository.^61^

The second voxel configuration was based on the hexagonal honeycomb array of the mesh design to characterize the flow within the pore of each mesh. Each voxel spanned an angle of 60° and a depth of 5 mm. The mean vertical, radial and azimuthal velocities were calculated for each voxel and used to plot the spatial distribution of vertical velocity of the tracer particle for each design, with vectors representing the direction of flow in the horizontal direction parallel to the mesh.

## Results and Discussion

### Flow Properties

**Figure 5:**
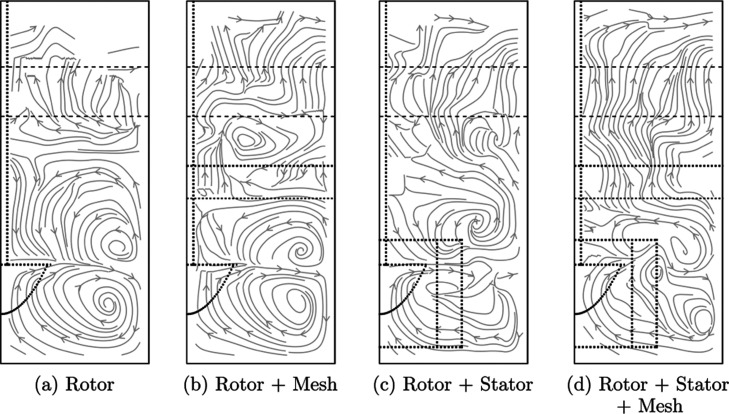
Particle flow pathlines from PEPT measurements of a hydrophobic tracer particle for each design: (a) rotor, (b) rotor + mesh, (c) rotor + stator and (d) rotor + stator + mesh. The horizontal axis of each azimuthal slice corresponds to the radial position 0 ⩽ *r* < 90 mm, and the vertical axis is the vertical position −60 ⩽ *z* < 160 mm; refer to Figure S1 for the geometry of the voxel configuration. The lip and approximate interface levels are indicated with dashed lines, and the impeller, stator and mesh are indicated with dotted lines.

Figure 5 shows the flow pathlines of a hydrophobic particle for different combinations of both retrofit designs studied. The rotor impeller (Figure 5(a)) produces an axial mixing pattern associated with two loops. There is a clear boundary at the pulp–froth interface that separates the lower axial mode of flow from upward rising pathlines in the froth. This separation suggests that the pulp is decoupled from the froth as per Mesa et al.^2^ and Norori-McCormac.^47^ The rotor + mesh combination (Figure 5(b)) has compressed mixing loops originating from the impeller, with some flow across the surface of the lower face of the mesh. There is an additional vortex above the mesh, possibly causing the recycling of material that has dropped back from the froth as per Mackay.^49^ In the case where only the stator is retrofitted (Figure 5(c)), the upper and lower axial mixing vortices are reduced in height, suggesting that turbulence is localized to the mixing mechanism and the upper pulp zone is correspondingly more quiescent. The center of the tank had slow moving flow with reduced circulation to the rest of the tank, and there is an additional mixing loop under the interface near the tank wall. The addition of the mesh to this system to form the rotor + stator + mesh combination (Figure 5(d)) increases the speed of flow through the mesh, which is directly upward and toward the froth. Most pathlines overflow the lip with this design, which suggests that the mesh increased the metallurgical recovery of the tank.

Additional plots of azimuthal slices of the velocity components in the radial, angular and vertical dimensions and the magnitude of velocity are given in the Supporting Information, Figures S3–S6. Also included are linear plots of the profile of velocity components with height for different radial positions in Figures S7–S9.

### Residence Time Distribution

**Figure 6:**
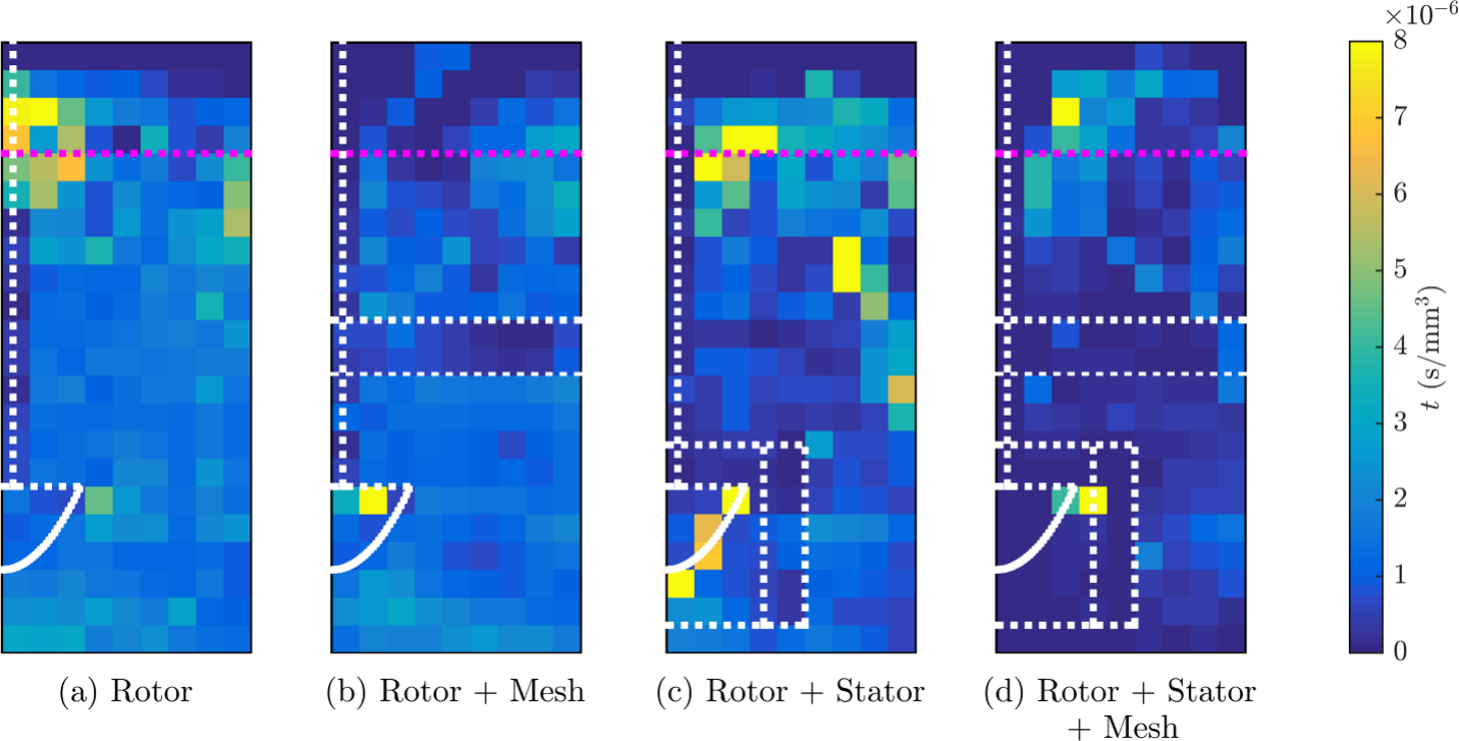
Residence time fraction per mm^3^*T* of the hydrophobic tracer particle from PEPT measurements with each design: (a) rotor, (b) rotor + mesh, (c) rotor + stator and (d) rotor + stator + mesh. The horizontal axis of each azimuthal slice corresponds to the radial position 0 ⩽ *r* < 90 mm, and the vertical axis is the vertical position −60 ⩽ *z* < 160 mm; refer to Figure S1 for the geometry of the voxel configuration. The lip level is indicated with a magenta dotted line, and the impeller, stator and mesh are indicated with white dotted lines.

Figure 6 shows the residence time distribution in an azimuthal slice calculated as a fraction relative to the total time the tracer particle spent in the tank during the test. High residence times near the impeller tip were measured for all four designs, which suggests that the tracer may have been caught up in between the impeller blades. In the center of the froth, high residence times were measured for the cases that used the rotor, rotor + stator and rotor + stator + mesh combinations (Figure 6), which evidenced a “dead zone” for the tracer associated with low circulation as the rising froth is not collected here. This dead zone in the froth has been described in previous works (e.g., Mesa et al.^2^ and Norori-McCormac^47^), and it appears to be characteristic of this type of laboratory tank. In an industrial tank, an internal launder, crowder or combination of both might be used to correct this issue.^5^ There was also high tracer residence time in the tank fitted with the rotor + stator configuration in the position of the additional mixing loop under the interface, which suggests an increase in entrainment or entrapment of the tracer particle with this configuration.

The addition of the mesh to the tank resulted in lower residence times in both cases. In the case of the rotor + mesh configuration (Figure 6(c)), the residence time was consistently low at positions above the mesh and at its lowest in the region of the potential secondary recirculation loop. This low residence time in the froth near the center of the tank implied that the additional loop moved material from the center of the tank back toward the lip, which suggests that the mesh helped to minimize the amount of solid material reaching the center of the froth, where it cannot be recovered without an internal launder or crowder. In the case of the rotor + stator + mesh configuration (Figure 6(d)), the addition of the mesh led to a lower residence time distribution through the pulp and froth, which is in agreement with the suggestion of a higher air recovery. A higher air recovery has been associated with higher metallurgical performance.^62^

### Flow through the Mesh

**Figure 7:**
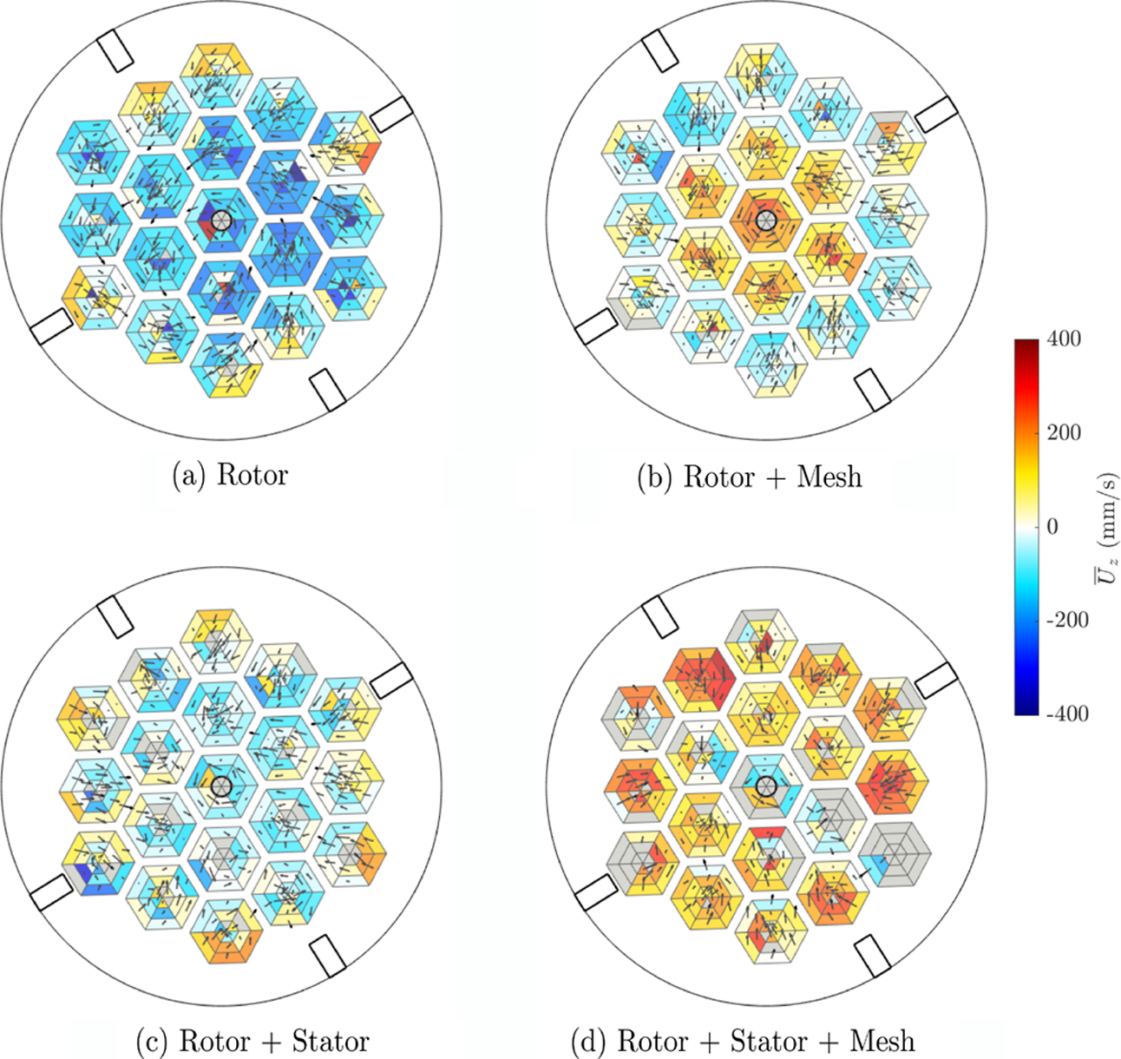
Average vertical particle velocity *U*_*z*_ and quiver plot of the horizontal velocity of a hydrophobic tracer particle derived from PEPT measurements in voxels aligned with the pores of the mesh insert at the lower level of the mesh. Retrofit design modifications were included: (a) rotor, (b) rotor + stator, (c) rotor + mesh, (d) rotor + stator + mesh. Voxels shaded gray did not have any data recorded.

Figure 7 shows the vertical velocity of the tracer particle in the lower 5 mm height of the mesh, with a quiver plot showing the horizontal flow profile. The data have been analyzed and presented using a voxel regime corresponding to the pores of the mesh to aid a direct comparison between designs, regardless of whether the mesh was present.

**Figure 8:**
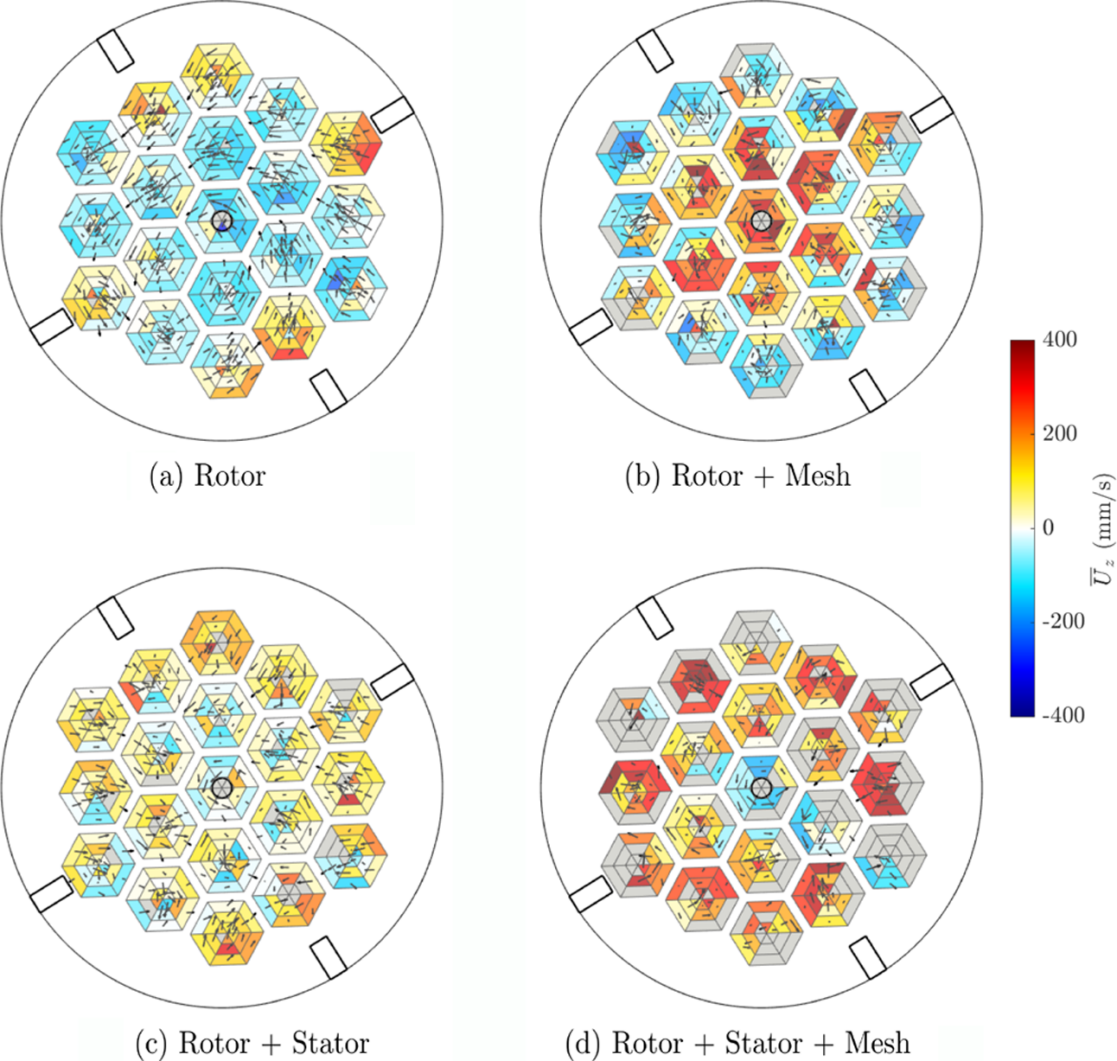
Average vertical particle velocity *U*_*z*_ and quiver plot of the horizontal velocity of a hydrophobic tracer particle derived from PEPT measurements in voxels aligned with the pores of the mesh insert at the upper level of the mesh. Retrofit design modifications were included: (a) rotor, (b) rotor + stator, (c) rotor + mesh, (d) rotor + stator + mesh. Voxels shaded gray did not have any data recorded.

Figure 7(a), with the rotor impeller only, shows that there was considerable swirl or rotational motion visible around the circumference of the vessel, and it was only near the vessel walls that the particle flow was directed to move upward toward the froth. In the case using the rotor + stator configuration (Figure 7(c)), the downward flow in the center was of reduced magnitude, with a similar profile to the rotor only case, of upward velocity near the walls. The quiver vectors tend to be directed inward toward the center of the vessel, in agreement with Figure 5(c). The addition of the mesh to the rotor system (Figure 7(b)) changed the general flow profile to upward in the center and downward at the outer ring of pores. The results with the rotor + stator + mesh combination of retrofits (Figure 8(d)) show the most pronounced difference in vertical velocity behavior, with the inclusion of both the stator and mesh retrofits leading to a generally net upward particle motion across the vessel. It is worth noting that the data set associated with this retrofit combination was smaller than the other three conditions as the tracer particle was broken during operation, and some voxels did not have any data recorded (shown as gray).

Where the mesh was present (Figure 7 (b,d)), the flow within the pores was generally outward from the center and rotating around the pore circumference near the edges of each pore, suggesting the geometry of each mesh pore influenced the length scale of macroturbulence in the mesh. In the central pore around the impeller shaft the flow was highly rotational, moving either upward with the rotor only (rotor + mesh combination in Figure 7(c)) or downward with the stator (rotor + stator + mesh combination in Figure 7(d)). As with the rotor + mesh configuration, rotational flow within each pore was visible in the flow profile of the rotor + stator + mesh combination; however, the vertical particle flow was also upward in the pores nearer the tank wall.

The addition of the mesh in both cases seems to break up the horizontal swirling effect near the pulp–froth interface, and instead introduces net upward motion, with rotational motion confined to the pores. This change in flow behavior could be associated with a higher recovery, as a result of lower froth residence times, and higher air recovery, as a result of more pathlines directed toward the lip.

Figure 8 shows an equivalent analysis of vertical velocity in the pores of the mesh for the upper level of the mesh, with a quiver plot of the horizontal velocity components. The general vertical particle flow profiles with different retrofits tend to be similar in appearance with the two levels of the mesh; however, the magnitudes are noticeably different with increasing height in the mesh.

Comparing results for the same retrofit combinations, Figures 7 and 8, starting with the rotor only in Figure 8(a), the vertical velocity tends to decrease with height in this region of the vessel, as the pulp becomes more quiescent with increasing distance from the impeller. The addition of the stator (rotor + stator in Figure 8(c)) leads to a positive vertical velocity in this region, in agreement with the different sizes of axial mixing vortices, as noted previously in Figure 5(c).

In both cases when the mesh is present (in Figure 8(b) rotor + mesh and (d) rotor + stator + mesh), the vertical tracer velocity tends to increase with height in the mesh. This is in agreement with increases in vertical velocity indicated in the higher spatial density of streamlines in the same region in Figure 5(b, d) and likely arises from restricting the flow volume of the vessel by adding the mesh. This increase in upward flow velocity of hydrophobic particles, coupled with the reduction of swirling motion at the interface, may explain the positive impact on froth stability and metallurgical recovery observed in previous studies using similar retrofit designs.^44,49^

On the scale of the pores of the mesh, with the rotor + mesh combination in Figure 8(b), there are pores with both regions of increasing and decreasing vertical velocity, which is more apparent with the higher velocity magnitudes in the upper region of the mesh. In addition, the quiver vectors in the central pores tend to have lower magnitude at the upper level of the mesh, suggesting a conversion of rotational kinetic energy to linear kinetic energy, which increases the upward particle velocity. These changes in particle velocity are less apparent with the rotor + stator + mesh combination, Figure 8(d), which may relate to the fewer number of voxel elements from a shorter experiment time. Overall, these features of the flow suggest a complex circulation behavior of particles within the pores and warrant future investigation of the particle flow in meshes with pores of different size.

### Turbulent Kinetic Energy

**Figure 9:**
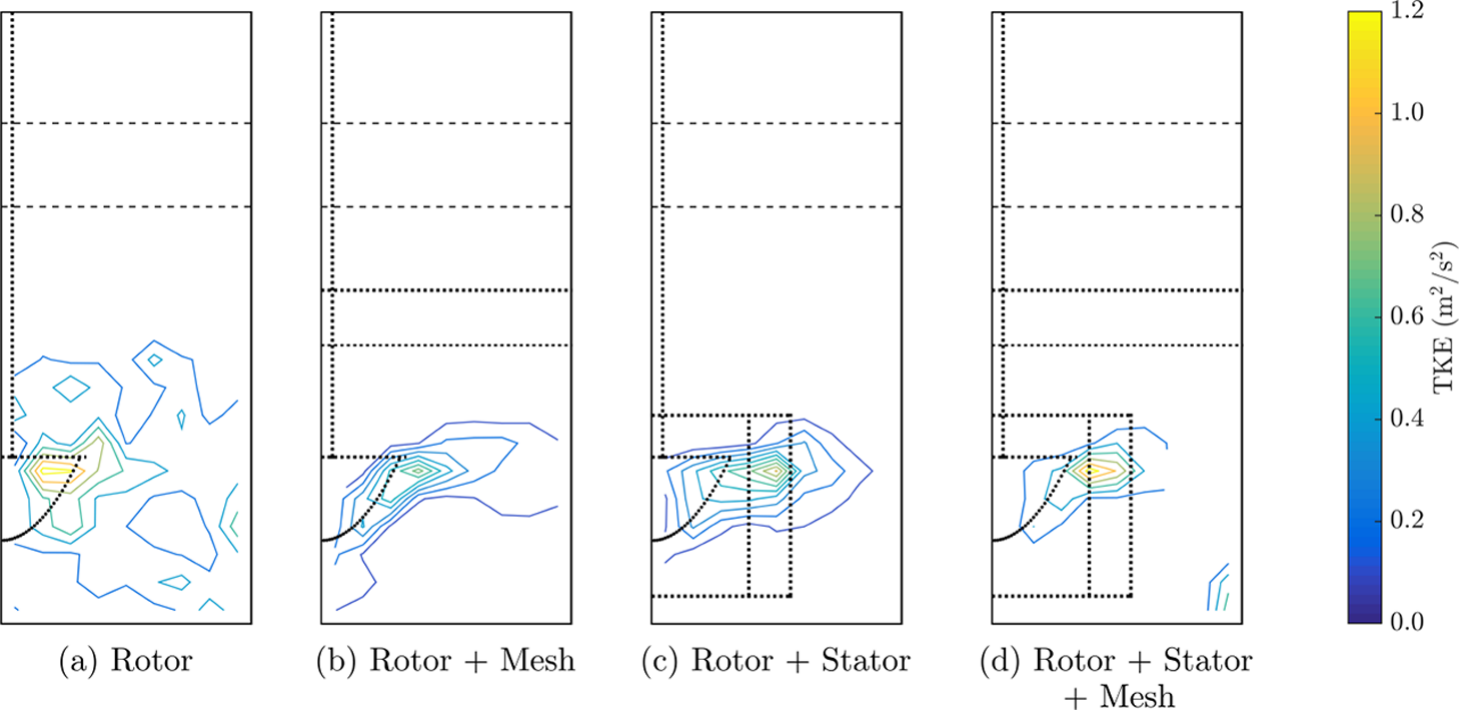
Contours of turbulent kinetic energy per unit mass (TKE) derived from PEPT measurements of a hydrophobic particle for each design: (a) rotor, (b) rotor + mesh, (c) rotor + stator and (d) rotor + stator + mesh. The horizontal axis of each azimuthal slice corresponds to the radial position 0 ⩽ *r* < 90 mm, and the vertical axis is the vertical position −60 ⩽ *z* < 160 mm; refer to Figure S1 for the geometry of the voxel configuration. The lip and approximate interface levels are indicated with dashed lines, and the impeller, stator and mesh are indicated with dotted lines.

Figure 9 shows the turbulent kinetic energy based on measurements of fluctuating velocity using PEPT for each design. The variances of the components of fluctuating velocity in the radial, azimuthal angular and vertical velocities are given in the Supporting Information, Figures S10–S12.

All four configurations show high turbulent kinetic energy in a region around the impeller. The TKE values obtained in these three phase experiments, reaching a peak of the order of 1 m^2^/s^2^ near the impeller, are consistent with previous experimental measurements in two-phase flotation systems^63^ and with the values used for bubble collision frequency simulations.^64^ This zone of high turbulent energy is the smallest in size for the case with the addition of the stator and mesh (rotor + stator + mesh combination in Figure 9(d)) and is largely confined to the stator. The largest zone was measured for the case using the rotor impeller without any retrofit design modification (Figure 9(a)), where turbulent energy was dissipated from the impeller across the width of the tank to the outer wall. Any particle–bubble aggregates that formed in the impeller discharge stream may have their particles detached in this broader turbulent zone.

In the Supporting Information, Figures S10–S12, it can be observed that the dominating component varies with the retrofit design. In the case of the rotor alone, the angular component of the TKE is dominant, while when the mesh + stator is installed, the predominant component is radial. This is related to previous findings^2^ that the stator considerably modifies the flow pattern, reducing pulp swirling and redirecting the impelled flow into a radial pattern. Also included are linear plots of the profile of TKE components with height for different radial positions in Figures S7–S9.

The addition of the mesh (rotor + mesh combination in Figure 9(b)) reduced the size of this turbulent zone, with higher turbulent kinetic energy extending immediately below the impeller and to the tank wall. The mesh may be introducing a downward back-pressure in the pulp, as suggested by the region of turbulent energy below the impeller in the case of the rotor + mesh configuration, and near the base of the tank for the rotor + stator + mesh configuration (Figure 9(b) and (d), respectively).

## Conclusions

This study used the PEPT technique to follow hydrophobic tracer particles in a laboratory scale flotation tank fitted with a rotor impeller mechanism and two retrofit design modifications: a stator and a horizontally positioned honeycomb mesh with a blocked section around the perimeter. The results show that the retrofit of a stator changed the flow pattern of the rotor impeller and restricted the dissipation of turbulent kinetic energy from the impeller discharge stream. The retrofit of a mesh to the tank reduced the horizontal swirling motion at the top of the pulp and redirected the flow to be upward and circulating around the pores of the mesh. The blocked section had a squeezing effect on the upward flow, tending to reduce the froth residence time and potentially improve the metallurgical performance of the tank. Suggestions have been made to the potential impact on flotation performance, which can only be verified in future test work.

## Supporting Information Available

Azimuthal slices of the vessel; particle size distribution; radial, azimuthal angular, and vertical velocities; magnitude of velocity; and contours of the radial, angular, and vertical components of the turbulent kinetic energy (PDF)
