# Supplementary material for: Effect of Retrofit Design Modifications on the Macroturbulence of a Three-Phase Flotation Tank—Flow Characterization Using Positron Emission Particle Tracking (PEPT)
Source: Ind Eng Chem Res. 2023 May 3;62(19):7580–91. doi: 10.1021/acs.iecr.2c04389 (PMC10202364; doi:10.1021/acs.iecr.2c04389)
Supplement: Supplementary file 1 — ie2c04389_si_002.pdf [file ie2c04389_si_002.pdf]

Supporting Information: The effect of retrofit design modifications on the macro-turbulence of a three-phase flotation tank – Flow characterisation using positron emission particle tracking (PEPT).

Katie Cole,[*,†,§] Diego Mesa,[*,‡,§] Michael van Heerden,[†,¶] and Pablo R. Brito-Parada[‡]

†*Department of Physics, University of Cape Town, Rondebosch, 7700, South Africa*

‡*Advanced Mineral Processing Research Group, Royal School of Mines, Imperial College London, South Kensington, London SW7 2BX, United Kingdom*

¶*iThemba LABS, Old Faure Rd, Eerste River, Cape Town, 7100, South Africa*

§*Contributed equally to this work*

E-mail: katie.cole@uct.ac.za; d.mesa@imperial.ac.uk

Additional figure representations

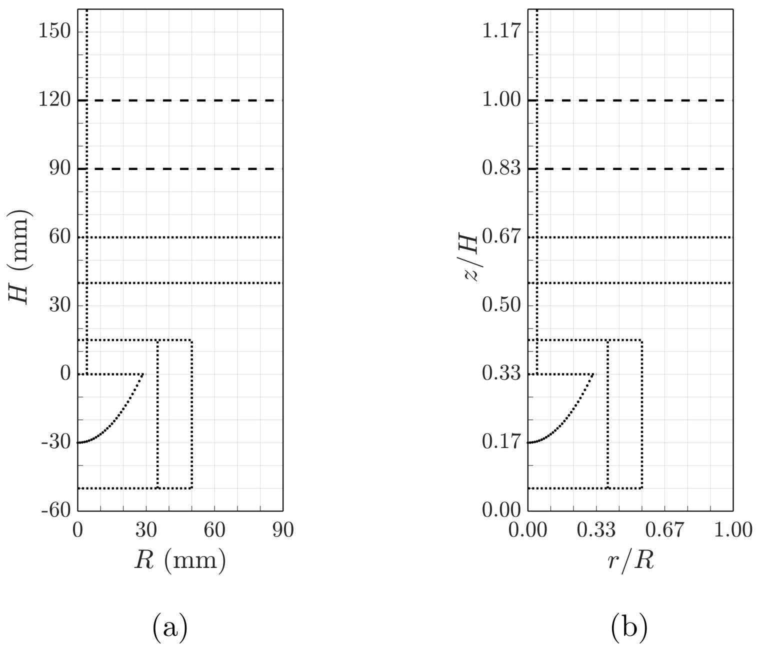

Figure S1: Key to figures of azimuthal slices of the vessel (a) the geometry in terms of vertical position z and radial position r and (b) the relative proportions of z to the vessel height H and r to vessel radius R. The voxels outlines are plotted relative to features of the vessel geometry including the rotor impeller, stator, mesh, interface and lip level. See the main article for further information.

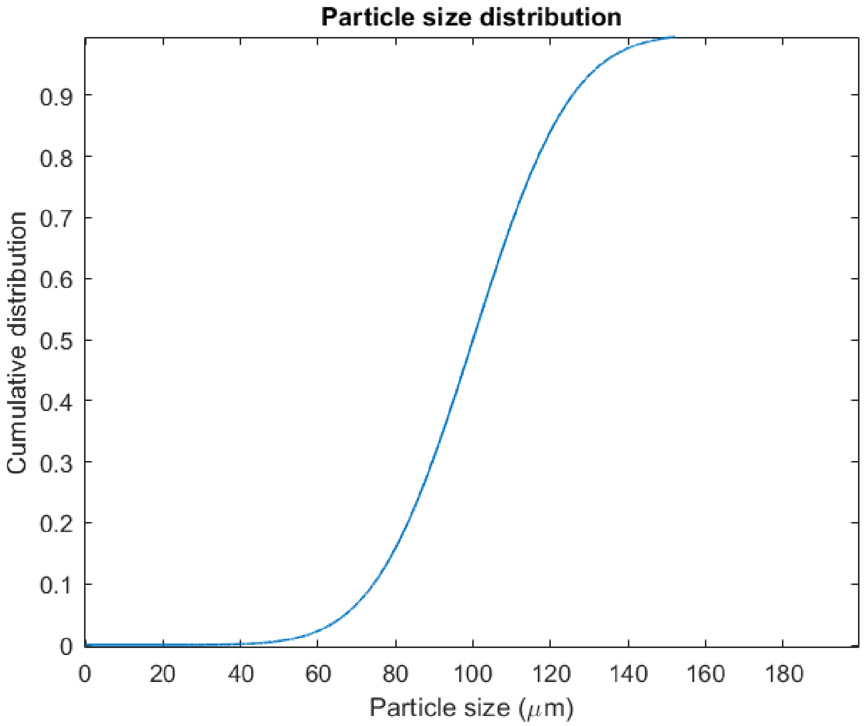

Figure S2: Particle size distribution of a sample of the silica solids in size +75 -150 μm used for flotation experiments. Measured using a Mastersizer 3000.

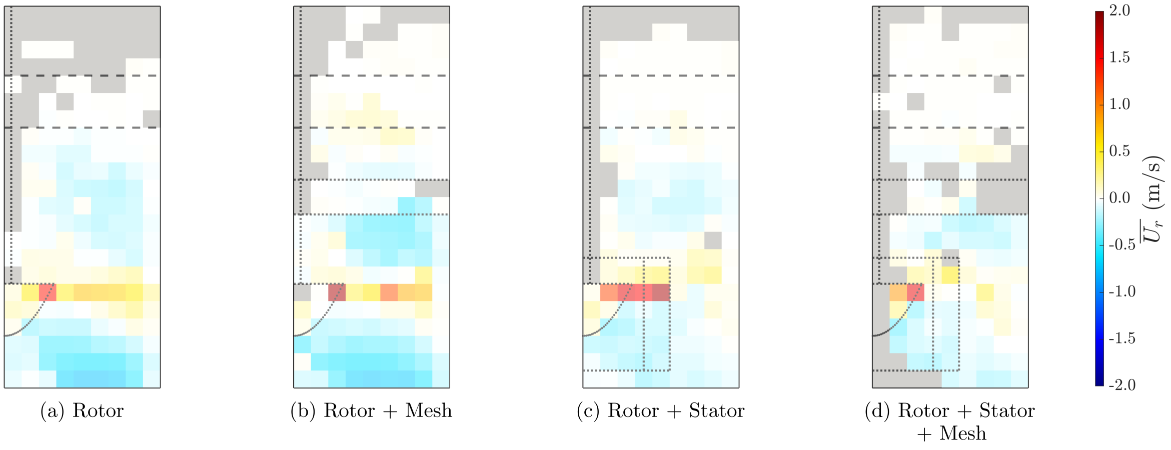

Figure S3: Radial velocity $\overline{U_r}$ from PEPT measurements of a hydrophobic tracer particle for each design: (a) rotor, (b) rotor + mesh, (c) rotor + stator and (d) rotor + stator + mesh. The horizontal axis of each azimuthal slice corresponds to the radial position $0 \leq r < 90$ mm and the vertical axis is the vertical position $-60 \leq z < 160$ mm; refer to Figure S1 for the geometry of the voxel configuration. The lip and approximate interface levels are indicated with dashed lines and the impeller, stator and mesh with dotted lines.

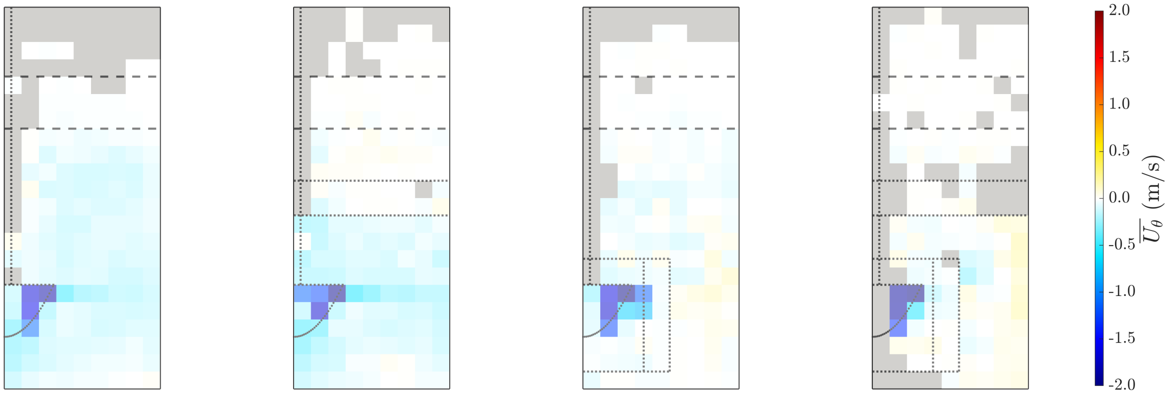

(a) Rotor (b) Rotor + Mesh (c) Rotor + Stator (d) Rotor + Stator + Mesh

Figure S4: Azimuthal angular velocity $\overline{U_\theta}$ from PEPT measurements of a hydrophobic tracer particle for each design: (a) rotor, (b) rotor + mesh, (c) rotor + stator and (d) rotor + stator + mesh. The horizontal axis of each azimuthal slice corresponds to the radial position $0 \leq r < 90$ mm and the vertical axis is the vertical position $-60 \leq z < 160$ mm; refer to Figure S1 for the geometry of the voxel configuration. The lip and approximate interface levels are indicated with dashed lines and the impeller, stator and mesh with dotted lines.

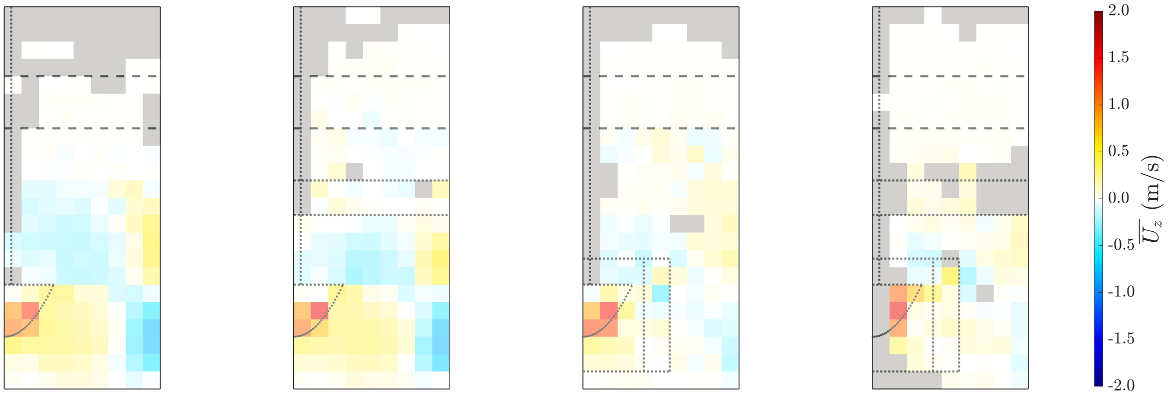

(a) Rotor (b) Rotor + Mesh (c) Rotor + Stator (d) Rotor + Stator + Mesh

Figure S5: Vertical velocity $\overline{U_z}$ from PEPT measurements of a hydrophobic tracer particle for each design: (a) rotor, (b) rotor + mesh, (c) rotor + stator and (d) rotor + stator + mesh. The horizontal axis of each azimuthal slice corresponds to the radial position $0 \leq r < 90$ mm and the vertical axis is the vertical position $-60 \leq z < 160$ mm; refer to Figure S1 for the geometry of the voxel configuration. The lip and approximate interface levels are indicated with dashed lines and the impeller, stator and mesh with dotted lines.

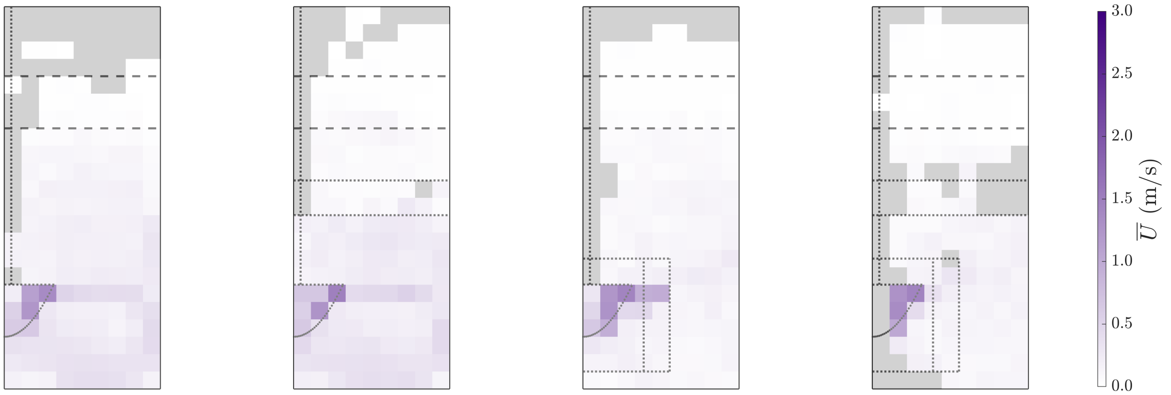

(a) Rotor

(b) Rotor + Mesh

(c) Rotor + Stator

(d) Rotor + Stator + Mesh

Figure S6: Magnitude of velocity $\overline{U}$ from PEPT measurements of a hydrophobic tracer particle for each design: (a) rotor, (b) rotor + mesh, (c) rotor + stator and (d) rotor + stator + mesh. The horizontal axis of each azimuthal slice corresponds to the radial position $0 \leq r < 90$ mm and the vertical axis is the vertical position $-60 \leq z < 160$ mm; refer to Figure S1 for the geometry of the voxel configuration. The lip and approximate interface levels are indicated with dashed lines and the impeller, stator and mesh with dotted lines.

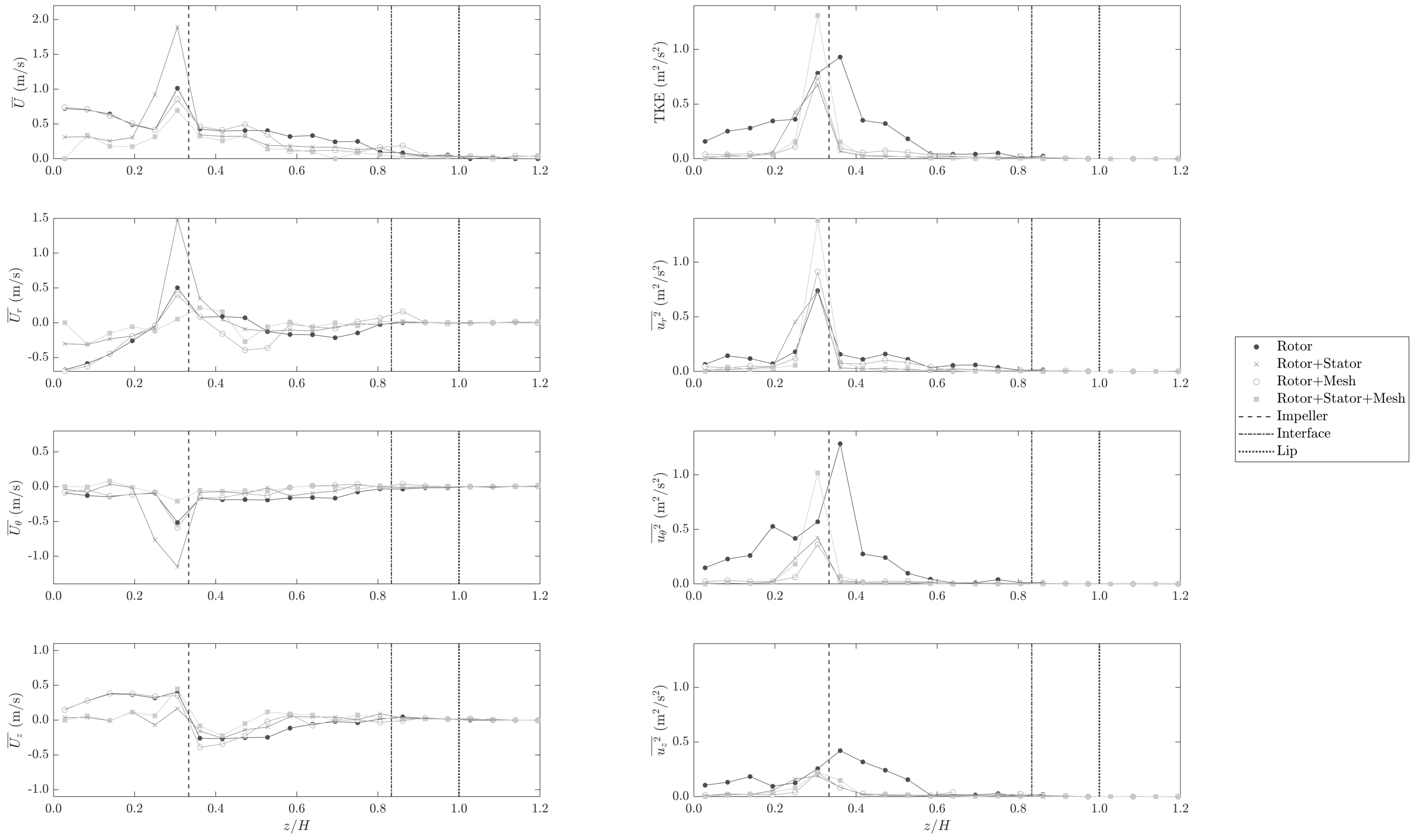

Figure S7: Plots of different velocity values of the hydrophobic tracer particle from PEPT measurements with vertical position z relative to the total height of the vessel H for a constant radial position r of $0.33 < r/R < 0.44$ relative to the radius of the vessel R. Subplots on the left from top to bottom are: the magnitude of the velocity $\overline{U}$, the radial velocity $\overline{U_r}$, the azimuthal angular velocity $\overline{U_\theta}$ and the vertical velocity $\overline{U_z}$, and on the right from top to bottom: the turbulent kinetic energy TKE, the variances of the fluctuating velocity component in the radial $\overline{u_r^2}$, azimuthal angular $\overline{u_\theta^2}$ and vertical $\overline{u_z^2}$ dimensions. Data are included for the four combinations of retrofit design modifications, rotor, rotor+stator, rotor+mesh and rotor+stator+mesh. The vertical positions of key features of the geometry of the vessel are included, the impeller, the interface and lip.

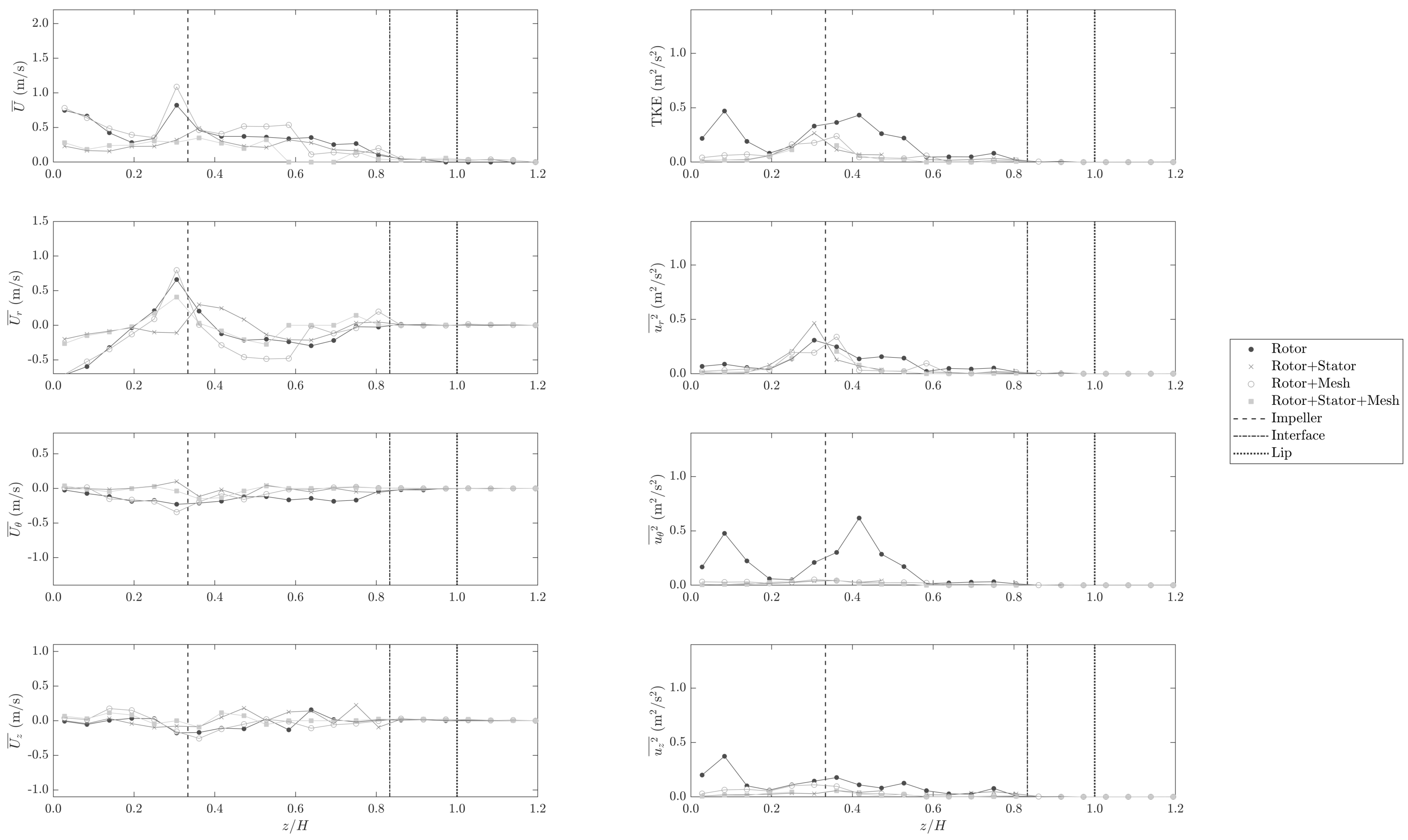

Figure S8: Plots of different velocity values of the hydrophobic tracer particle from PEPT measurements with vertical position z relative to the total height of the vessel H for a constant radial position r of $0.67 < r/R < 0.78$ relative to the radius of the vessel R. Subplots on the left from top to bottom are: the magnitude of the velocity $\overline{U}$, the radial velocity $\overline{U_r}$, the azimuthal angular velocity $\overline{U_\theta}$ and the vertical velocity $\overline{U_z}$, and on the right from top to bottom: the turbulent kinetic energy TKE, the variances of the fluctuating velocity component in the radial $\overline{u_r{}^2}$, azimuthal angular $\overline{u_\theta{}^2}$ and vertical $\overline{u_z{}^2}$ dimensions. Data are included for the four combinations of retrofit design modifications, rotor, rotor+stator, rotor+mesh and rotor+stator+mesh. The vertical positions of key features of the geometry of the vessel are included, the impeller, the interface and lip.

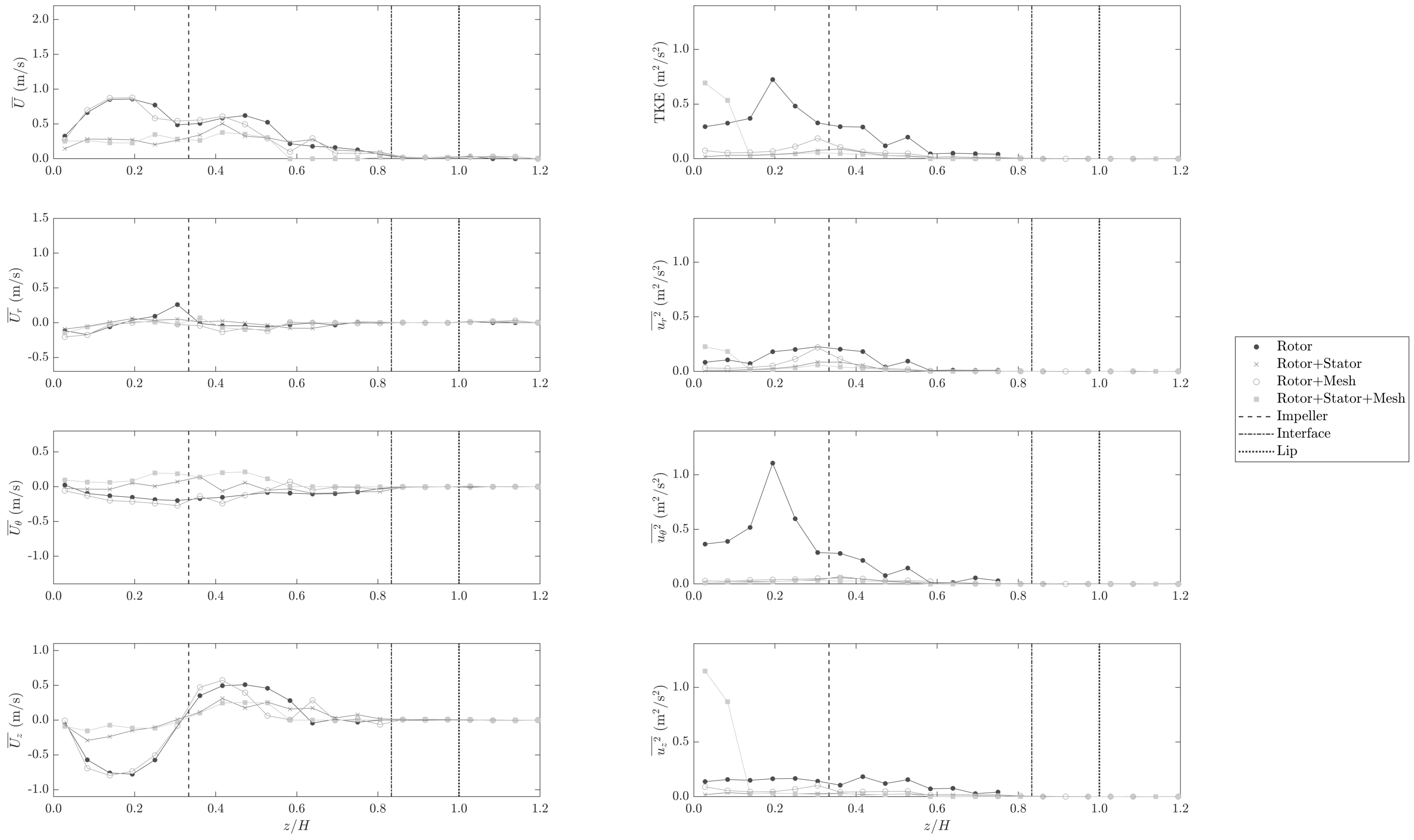

Figure S9: Plots of different velocity values of the hydrophobic tracer particle from PEPT measurements with vertical position z relative to the total height of the vessel H for a constant radial position r of $0.89 < r/R < 1.00$ relative to the radius of the vessel R. Subplots on the left from top to bottom are: the magnitude of the velocity $\overline{U}$, the radial velocity $\overline{U_r}$, the azimuthal angular velocity $\overline{U_\theta}$ and the vertical velocity $\overline{U_z}$, and on the right from top to bottom: the turbulent kinetic energy TKE, the variances of the fluctuating velocity component in the radial $\overline{u_r{}^2}$, azimuthal angular $\overline{u_\theta{}^2}$ and vertical $\overline{u_z{}^2}$ dimensions. Data are included for the four combinations of retrofit design modifications, rotor, rotor+stator, rotor+mesh and rotor+stator+mesh. The vertical positions of key features of the geometry of the vessel are included, the impeller, the interface and lip.

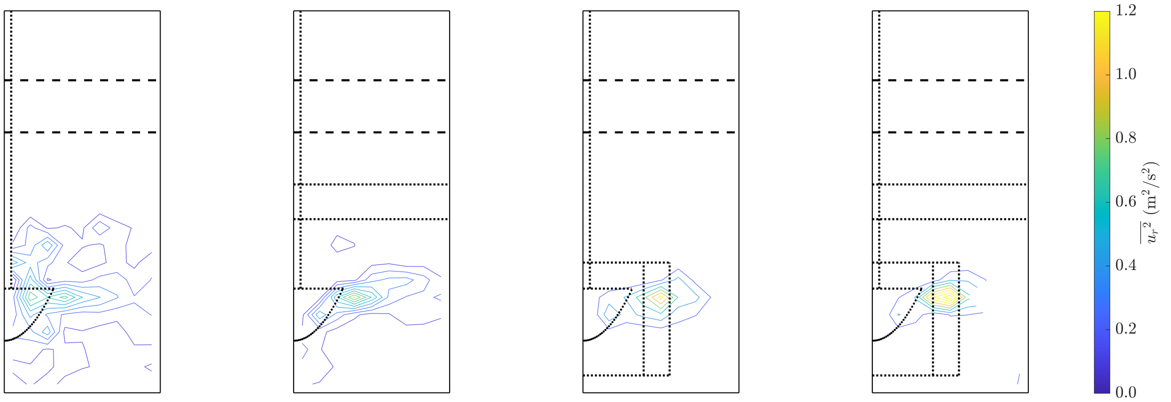

(a) Rotor (b) Rotor + Mesh (c) Rotor + Stator (d) Rotor + Stator + Mesh

Figure S10: Contours of the radial component of the turbulent kinetic energy per unit mass ($\overline{u_r{}^2}$) derived from PEPT measurements of a hydrophobic particle for each design: (a) rotor, (b) rotor + mesh, (c) rotor + stator and (d) rotor + stator + mesh. The lip and approximate interface levels are indicated with dashed lines and the impeller, stator and mesh with dotted lines.

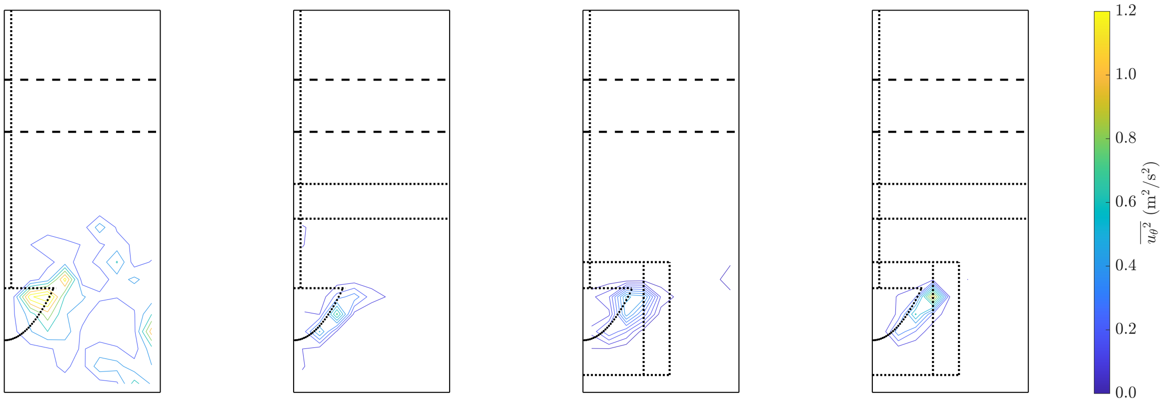

(a) Rotor (b) Rotor + Mesh (c) Rotor + Stator (d) Rotor + Stator + Mesh

Figure S11: Contours of the angular component of the turbulent kinetic energy per unit mass ($\overline{u_\theta{}^2}$) derived from PEPT measurements of a hydrophobic particle for each design: (a) rotor, (b) rotor + mesh, (c) rotor + stator and (d) rotor + stator + mesh. The lip and approximate interface levels are indicated with dashed lines and the impeller, stator and mesh with dotted lines.

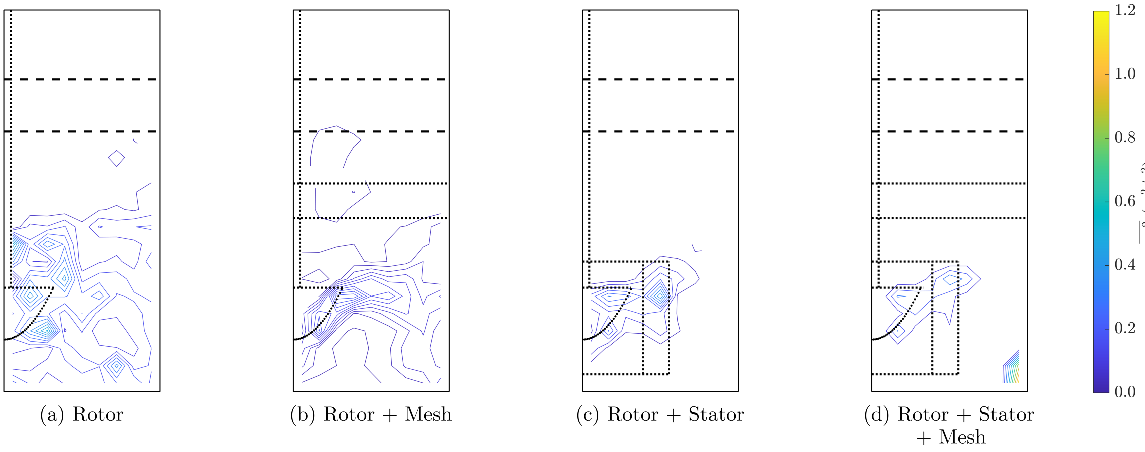

Figure S12: Contours of the vertical component of the turbulent kinetic energy per unit mass ($\overline{u_z{}^2}$) derived from PEPT measurements of a hydrophobic particle for each design: (a) rotor, (b) rotor + mesh, (c) rotor + stator and (d) rotor + stator + mesh. The lip and approximate interface levels are indicated with dashed lines and the impeller, stator and mesh with dotted lines.
